# Preferences for pre‐exposure prophylaxis delivery via online pharmacy among potential users in Kenya: a discrete choice experiment

**DOI:** 10.1002/jia2.26356

**Published:** 2024-10-09

**Authors:** Enrique M. Saldarriaga, Yilin Chen, Michalina A. Montaño, Nicholas Thuo, Catherine Kiptinness, Fern Terris‐Prestholt, Andy Stergachis, Melissa Latigo Mugambi, Kenneth Ngure, Katrina F. Ortblad, Monisha Sharma

**Affiliations:** ^1^ The Comparative Health Outcomes, Policy, and Economics (CHOICE) Institute University of Washington Seattle Washington USA; ^2^ Vaccine and Infectious Diseases Division (VIDD) Fred Hutchinson Cancer Center Seattle Washington USA; ^3^ Department of Global Health University of Washington Seattle Washington USA; ^4^ Partners in Health and Research Development Centre for Clinical Research, Kenya Medical Research Institute Nairobi Kenya; ^5^ Department of Global Health and Development Faculty of Public Health and Policy, London School of Hygiene and Tropical Medicine London UK; ^6^ School of Public Health Jomo Kenyatta University of Agriculture and Technology Nairobi Kenya; ^7^ Public Health Science Division Fred Hutchinson Cancer Center Seattle Washington USA

**Keywords:** discrete choice experiment, Kenya, online delivery, pharmacies, preferences, PrEP

## Abstract

**Introduction:**

Oral pre‐exposure prophylaxis (PrEP) is highly effective, but coverage remains low in high HIV prevalence settings. Initiating and continuing PrEP remotely via online pharmacies is a promising strategy to expand PrEP uptake, but little is known about potential users’ preferences.

**Methods:**

We conducted a discrete choice experiment (DCE) to assess preferences for online pharmacy PrEP services. We partnered with MYDAWA, an online pharmacy in Nairobi, Kenya. Eligibility criteria were: ≥18 years, not known HIV positive, interested in PrEP. The DCE contained four attributes: PrEP eligibility assessment (online self‐assessed, guided), HIV test type (provider administered, oral HIV self‐test [HIVST], blood‐based HIVST), clinical consultation (remote, in‐person) and user support options (text messages, phone/video call, email). Additionally, participants indicated whether they were willing to uptake their selected service. The survey was advertised on MYDAWA's website; interested participants met staff in‐person at a convenient location to complete the survey from 1 June to 20 November 2022. We used conditional logit modelling with an interaction by current PrEP use to estimate overall preferences and latent class analysis (LCA) to assess preference heterogeneity.

**Results:**

Overall, 772 participants completed the DCE; the mean age was 25 years and 54% were female. Most participants indicated a willingness to acquire online PrEP services, with particularly high demand among PrEP‐naive individuals. Overall, participants preferred remote clinical consultation, HIV self‐testing, online self‐assessment and phone call user support. The LCA identified three subgroups: the “prefer online PrEP with remote components” group (60.3% of the sample) whose preferences aligned with the main analysis, the “prefer online PrEP with in‐person components” group (20.7%), who preferred in‐person consultation, provider‐administered HIV testing, and guided assessment, and the “prefer remote PrEP (18.9%)” group who preferred online PrEP services only if they were remote.

**Conclusions:**

Online pharmacy PrEP is highly acceptable and may expand PrEP coverage to those interested in PrEP but not accessing services. Most participants valued privacy and autonomy, preferring HIVST and remote provider interactions. However, when needing support for questions regarding PrEP, participants preferred phone/SMS contact with a provider. One‐fifth of participants preferred online PrEP with in‐person components, suggesting that providing multiple options can increase uptake.

## 1 INTRODUCTION

Pre‐exposure prophylaxis (PrEP) is a highly effective HIV prevention strategy but scale‐up has fallen short of targets, with only 10% of those at HIV risk globally using PrEP [1]. In Kenya and other Eastern and Southern Africa (ESA) countries, PrEP is mainly delivered through clinics; barriers contributing to low uptake and persistence include long travel and wait times, privacy concerns, limited hours of operation, stigma and understaffing [2, 3, 4, 5]. Differentiated service delivery models are needed to overcome these barriers and achieve high PrEP uptake and retention.

PrEP provision *via* online pharmacies is a novel strategy that could expand coverage by offering convenient, private and user‐centred services. Online PrEP consists of remote clinician consultations, community‐based HIV testing, PrEP drug delivery and virtual user support. The growing availability of telehealth services in ESA, which expanded further during COVID‐19, can provide a platform for direct‐to‐client distribution of health products, digital health education, and virtual provider interactions. Coupled with Kenya's national goal of increasing PrEP coverage, digital health systems are a promising strategy to facilitate PrEP rollout by overcoming structural barriers associated with current delivery [6].

Understanding client preferences is crucial for tailoring PrEP strategies to optimize uptake, adherence and retention. Discrete choice experiments (DCEs) are an increasingly popular method to quantitatively assess preference for healthcare services [7]. Grounded in economic theory, DCEs evaluate how individuals value different components of a service to identify the most important attributes and understand trade‐offs between components. DCEs are particularly useful in evaluating preferences for novel services for which observed uptake data are not available. We sought to assess preferences for obtaining PrEP through an online pharmacy among potential users in Kenya. To our knowledge, this is the first study to evaluate client preferences for online PrEP delivery.

## 2 METHODS

### 2.1 Population and setting

The DCE protocol has been previously published [8]. Briefly, the study was a collaboration between researchers at the University of Washington, MYDAWA: Kenya's first licensed online pharmacy (https://mydawa.com), and Partners in Health and Research Development: a Kenya‐based organization. The target population was selected to closely mirror likely users of online PrEP. Eligible individuals were aged ≥18 years, not known to have HIV, interested in PrEP and able/willing to provide informed consent. Participants were recruited from 1 June to 20 November 2022, *via* banner ads on MYDAWA website pages displaying HIV self‐tests (HIVSTs) and through flyers mailed with client's HIVST purchases. Interested participants who called the advertised study phone number were screened for eligibility and interviewers arranged a meeting at a convenient location to administer the survey *via* tablet in participants’ preferred language (English or Kiswahili). Participants were reimbursed 1000 KES (∼$8.70 USD). We used Research Electronic Data Capture for screening and consent data [9].

This study was approved by the University of Washington (STUDY00014011), Scientific and Ethics Review Unit at the Kenya Medical Research Institute—(KEMRI/SERU/4364) and Nairobi Metropolitan Services (EOP/NMS/HS/128).

### 2.2 DCE development

Initial attributes and levels were developed in collaboration with the Kenyan Ministry of Health, NGOs and professional organizations, and based on a care pathway for online PrEP services in Kenya [10]. The main components include: (1) PrEP eligibility assessment: based on a Rapid Assessment Screening Tool used in Kenya to assess HIV risk for PrEP eligibility; (2) HIV testing: to ensure individuals are HIV negative prior to initiating PrEP; (3) clinical consultation: medical assessment to ensure clients can safely take PrEP; (4) PrEP delivery: PrEP delivered to clients’ preferred location; and (5) PrEP support: ongoing support for client questions/concerns questions about PrEP. Attributes and levels were refined after eight in‐depth interviews with experts in HIV prevention, PrEP delivery and representatives from MYDAWA. The final list included: PrEP eligibility assessment (self‐assessment or guided by clinical provider), HIV test type (blood‐based HIVST, oral HIVST, provider administered HIV test), clinical consultation (remote or in‐person) and user support (SMS, phone, or email) (Table 1). Attribute descriptions and images were pre‐tested in cognitive interviews to ensure clarity and cultural appropriateness [8]. For example, the cell phone image was modified to look like phones most commonly used in this setting.

**Table 1:** Attributes and levels in the discrete choice experiment

Attribute and description	Levels	Illustrative image
PrEP eligibility assessment: *Method for conducting client eligibility assessment for PrEP*	Online self‐assessment using screening questions (phone number in case of questions)	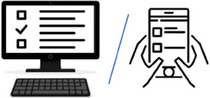
	Guided assessment with a remote clinical provider (via a phone call or WhatsApp)	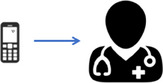
HIV test type: *Type of HIV test delivered for PrEP initiation*	Oral fluid HIV self‐test (at setting of your choice)	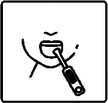
	Blood‐based HIV self‐test (at setting of your choice)	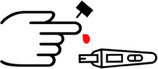
	Healthcare provider administers HIV test at setting of your choice (blood‐based)	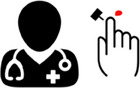
Clinical consultation for prescribing PrEP: *Consultation needed to prescribe PrEP*	Remote clinical consultation with provider (via a phone call or video chat)	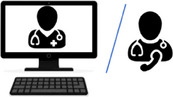
	In‐person clinical consultation with provider after completing HIV test (at a setting of your choice)	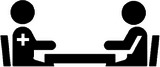
User support options for PrEP: *Method for discussing your questions for PrEP with healthcare provider*	SMS	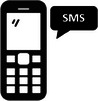
	Phone/video call	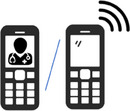
	Email	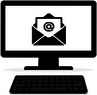

### 2.3 Survey design

The final DCE was designed using Lighthouse Studio Version 9.8.1 (Sawtooth Software) and included five attributes, each with two or three levels [11]. Participants were presented with eight choice tasks to minimize cognitive burden while maintaining design efficiency [12]. In each task, participants were shown two hypothetical online PrEP delivery services and asked to choose their preferred one. This was followed by an opt‐out question: “If the PrEP service you just chose was available, do you think you would actually use it?” The dual format design [13] enabled us to gather information on participants’ preferences for PrEP services and willingness to uptake the service (see Appendix S1 for example choice task). We used a D‐efficient design to identify a balanced subset of comparisons that maximizes trade‐offs between attribute‐levels to optimize the information gathered [14]. We estimated a minimal sample size of 700 to minimize uncertainty in the estimated coefficients and enable the evaluation of preference heterogeneity [15].

The survey first provided participants with a brief overview of DCEs and attributes and corresponding levels using text descriptions and images. Interviewers read each attribute description and paused to answer questions before moving to the next description. Participants were then presented with the DCE questions. Lastly, participants answered questions about demographics: history of PrEP use, knowledge and eligibility for PrEP, HIV testing, interest and use of online pharmacies, sexual behaviours, online PrEP acceptability and maximum stated willingness to pay (WTP) for different components of online PrEP services (Table 2). To evaluate the acceptability of online PrEP services, we included validated questions measuring various component constructs of acceptability (including self‐efficacy, burden and perceived effectiveness), defined in the Theoretical Framework of Acceptability (TFA) [16]. The TFA is commonly used in the context of HIV interventions defines acceptability as a multi‐faceted construct and evaluates the extent to which individuals consider a health intervention to be appropriate, based on anticipated or experienced responses to the intervention [16].

**Table 2:** Characteristics of DCE questionnaire respondents, *n*/*N* (%)

DCE participants (*N* = 772)	*n* (%)
**Demographic characteristics**	
Age	
18−24 years	333 (43.1)
≥25 years	439 (56.9)
Age, years (median, IQR)	25 (22–29)
Gender	
Male	345 (44.7)
Female	418 (54.1)
Other	9 (1.2)
Education	
Primary	21 (2.7)
Secondary	373 (48.3)
Technical or vocational school	174 (22.5)
University or higher	204 (26.4)
Employment^a^	
Employed, full‐time	177 (22.9)
Employed, part‐time	258 (33.4)
Employed, seasonal	127 (16.5)
Employed, multiple jobs	33 (4.3)
Not employed	168 (21.8)
Monthly income,^b^ median in KSH (IQR)	10000 (5000–20,000)
**HIV, PrEP and online pharmacy engagement**	
Ever tested for HIV	768 (99.5)
Months since the most recent HIV test^c^	4 (1–9)
Ever used HIV self‐test	429 (55.6)
Heard of PrEP prior to study participation	750 (97.2)
Ever taken PrEP	300 (38.9)
Currently taking PrEP	143 (18.5)
Ever purchased products from an online pharmacy	190 (24.6)
**Sexual behaviour**	
Sexual partners, prior 3 months	
No sexual partners in the prior 3 months	85 (11)
1 primary partner and no other partners	269 (34.8)
1 primary and 1+ casual partner(s)	238 (30.8)
1+ casual partner(s)	172 (22.3)
Number of sexual partners, prior 3 months; *n* = 658 (med, IQR)	2 (1–3)
Sex without a condom, prior 6 months	615 (79.7)
Exposure to HIV, prior 6 months	249 (32.3)
Diagnosis or treatment of STI, prior 6 months	106 (13.7)
**Maximum stated WTP for components of online PrEP delivery**	**Median in KSH (IQR)**
Blood‐based HIV self‐test	200 (100–300)
Oral HIV self‐test	200 (100–300)
Remote clinical consultation	300 (150–500)
One‐month supply of PrEP pills	300 (200–500)
Delivery of PrEP pills	200 (100–200)
Maximum stated WTP for package of PrEP delivery services^d^	1000 (800–1975)
**Acceptability for online PrEP delivery**	
How much would you like or dislike obtaining PrEP through an online platform?	
Strongly dislike	6 (0.8)
Somewhat dislike	5 (0.6)
Somewhat like	100 (12.9)
Strongly like	651 (84.4)
No opinion	10 (1.3)
How much effort do you think would take you to obtain PrEP online?	
No effort	164 (21.2)
A little effort	425 (55.1)
A moderate effort	145 (18.8)
A huge amount of effort	36 (4.7)
No opinion	2 (0.3)
How confident do you feel about your ability to navigate an online website to obtain PrEP?	
Very unconfident	9 (1.2)
Somewhat unconfident	9 (1.2)
Somewhat confident	95 (12.3)
Very confident	656 (84.9)
No opinion	3 (0.4)
How much do you agree or disagree with the following statement: Online PrEP delivery would help reduce HIV in my community?	
Strongly disagree	12 (1.6)
Somewhat disagree	16 (2.1)
Somewhat agree	171 (22.1)
Strongly agree	570 (73.8)
No opinion	3 (0.4)

Abbreviations: IQR, inter‐quartile range; KSH, Kenyan shillings; PrEP, pre‐exposure prophylaxis; STI, sexually transmitted infection; WTP, willingness to pay.

^a^ Nine participants did not answer this question.

^b^ One‐hundred and one participants did not answer this question (*n* = 671).

^c^ Eight participants did not answer this question (*n* = 764).

^d^ Package of PrEP delivery service includes HIV testing, remote clinical consultation, 1‐month supply of PrEP drugs and delivery.

### 2.4 Statistical analysis

We fitted effects‐coded choice data to a conditional logit model to estimate preference weights (PW) for each attribute‐level, including an interaction term for the opt‐out by current PrEP use to assess demand among those already accessing PrEP versus non‐users [17]. With effects coding, estimates for each level represent preference for that level relative to the mean attribute effect and the omitted level is estimated by the negative sum of other included levels. Within an attribute, positive PWs indicate greater preference for a level compared to others, and negative PWs indicate lower preference. The relative importance of an attribute in participants’ decision‐making was quantified as the absolute difference between the most and least‐preferred levels to obtain the attribute's range of influence, and then divided by the sum of all other attributes ranges [18].

We conducted latent class analysis (LCA) to assess preference heterogeneity [19]. We fitted LCA models with 2–5 classes and determined the optimal number of classes based on goodness of fit, using the adjusted Akaike and Bayesian Information Criteria, and whether adding another class improved understanding of the sample's preference heterogeneity. We included individual‐level characteristics in LCA models to quantify associations of class membership based on prior PrEP literature [2, 3, 20, 21], including age, sex, education, employment, known HIV exposure in ≥6 months, past purchases from an online pharmacy and current PrEP use. Since current PrEP usage was added to class membership, each class had one opt‐out coefficient instead of two, as in the previous analysis. We conducted analyses in Stata version 14.0. We used *lclogit2* for LCA estimation and *lclogitml2* for standard errors.

## 3 RESULTS

### 3.1 Participant characteristics

**Figure 1:**
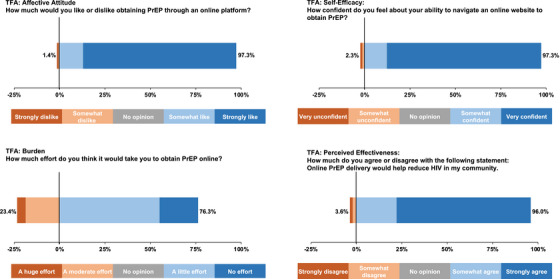
Acceptability of online PrEP delivery. Abbreviations: PrEP, pre‐exposure prophylaxis; TFA, theoretical framework of acceptability.

A total of 772 participants completed the survey. Participants were young: mean age of 25 years (IQR: 22–29) and slightly more than half were female (54%) (Table 2). Participants generally had high education levels (70% completed secondary or vocational school and an additional 26% completed university or higher) and most were employed (77%). Nearly all (99.5%) tested for HIV previously, with a median of 4 months since last test (IQR: 1–9); 56% had used HIVST. A high proportion of participants reported behaviours associated with HIV risk: 53% reported multiple partners in the past 3 months, 32% had a known HIV exposure in the past 3 months and 14% were treated for a sexually transmitted infection (STI). Overall, 39% reported ever taking PrEP; 19% were currently taking PrEP; and 25% of participants had purchased products from an online pharmacy. The maximum stated WTP for the package of PrEP services was 1000 KES (IQR: 800–1975; ∼$7 USD) (Table 2). Online PrEP was highly acceptable; 85% of participants strongly liked it and 86% reported confidence in their ability to navigate a website to obtain PrEP (Table 1 and Figure 1).

### 3.2 Participant preferences

**Figure 2:**
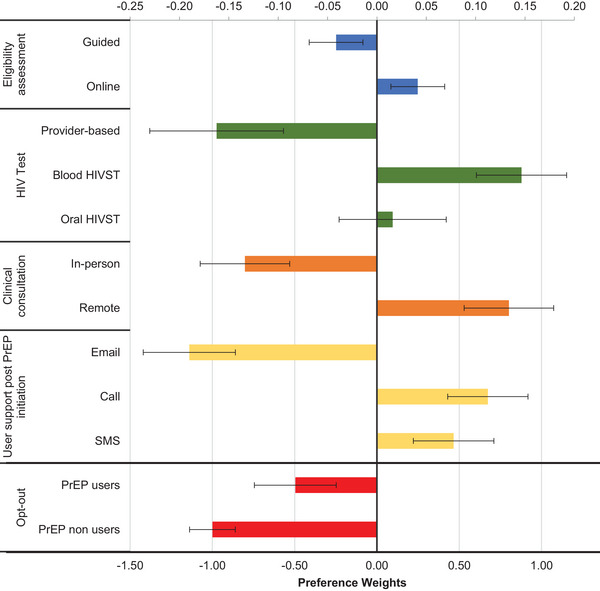
Estimated preference weights for an online PrEP‐delivery service in Kenya. Results from the discrete choice experiment for the overall sample opt‐out coefficients presented in separate (bottom) axis for better visualization. Abbreviation: PrEP, pre‐exposure prophylaxis. The opt‐out coefficient was based on participants’ responses to the question, “Would you choose to get PrEP using this service if it were available?”

Figure 2 displays the main analysis evaluating participant preferences with an interaction term for opt‐out by current PrEP use. The opt‐out (i.e. not choosing online PrEP) had negative PWs for both groups, with PrEP non‐users showing a larger negative preference than current users. All four components of online PrEP service influenced participant preferences, with HIV test type and user support being the two most important (32% and 31% relative importance, respectively, Appendix S2), followed closely by consultation (27%) and lastly clinical assessment with a small relative impact (9%).

For HIV testing, participants preferred blood‐based HIVST (0.15, 95% CI 0.1, 0.19); oral HIVST was less preferred (0.02, 95% CI −0.04, 0.07) and provider‐administered HIV testing had a negative PW (−0.16, 95% CI −0.23, −0.09). For user support, participants had a slight preference for phone call (0.11, 95% CI 0.07, 0.15) over SMS (0.08, 95% CI 0.04, 0.12), while email had a negative PW (−0.19, 95% CI −0.24, −0.14). For clinical consultation to ensure PrEP can be safely used, participants strongly preferred remote (0.13, 95% CI 0.09, 0.18) over in‐person consultation. Finally, for PrEP eligibility assessment, online self‐assessment was preferred (0.04, 95% CI 0.01, 0.07) over a provider conducting the assessment. Overall, participants greatly preferred remote over in‐person or provider‐guided services, except for user support, where they preferred phone calls to answer PrEP‐related questions.

### 3.3 Preference heterogeneity

**Figure 3:**
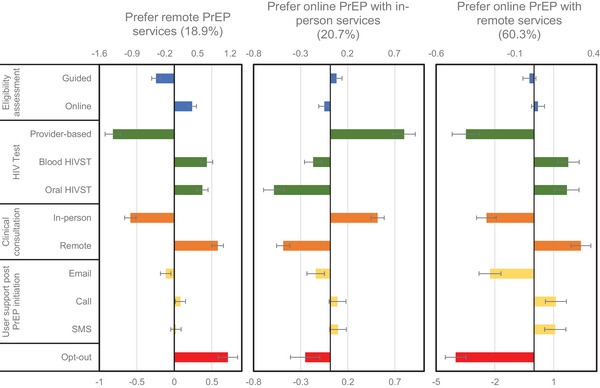
Estimated preference weights for an online PrEP‐delivery service in Kenya. Abbreviation: PrEP, pre‐exposure prophylaxis. Results from the latent class analysis for three classes. Opt‐out coefficients are presented in a separate (bottom) axis, for ease of visualization. Attributes assessed include both in‐person and remote services, for example guided clinical assessment, provider‐administered HIV testing and in‐person clinical consultation are considered in‐person services, while online‐self assessment, HIVST and remote clinical consultation are considered remote services. The opt‐out coefficient was based on participants’ responses to the question, “If the PrEP service you just chose was available, do you think you would actually use it?”

We found three classes had the best goodness of fit and provided the most meaningful partition in our sample. Classes were labelled post‐hoc as “prefer online PrEP with remote components” (60.3% of the sample), “prefer online PrEP with in‐person components” (20.7%) and “prefer remote PrEP (18.9%)” (Figure 3).

The “prefer online PrEP with remote components” group comprised most of the sample (60.3%). These participants greatly preferred online PrEP, reflected by a large negative opt‐out coefficient. Their preferences generally aligned with the main analysis: online self‐assessment, HIVST, remote clinical consultation and call or SMS user support; clinical assessment had the smallest impact on decision‐making. However, in contrast with the full sample, participants in this group did not have a preference between blood‐based and oral HIVST—both provided similarly positive utility. Class membership was more likely among those who previously used online pharmacies, were employed and not using PrEP (Table 3).

**Table 3:** Associations of class membership and participants’ characteristics, relative to Class 3

	Class 3: Prefer online PrEP with remote services Reference	Class 2: Prefer online PrEP with in‐person services		Class 1: Prefer remote PrEP services	
	%^a^	%	RRR^b^ (95% CI); *p*‐value	%	RRR (95% CI); *p*‐value
Age (median)	26	25	0.98 (0.95, 1.01); 0.161	25	0.97 (0.94, 1.01); 0.097
Sex					
Female	52	26	Ref	22	Ref
Male	44	36	1.54 (1.06, 2.24); 0.024	20	1.02 (0.68, 1.54); 0.925
Education level					
Above secondary	49	28	Ref	23	Ref
High‐school diploma or less	49	32	0.99 (0.67, 1.47); 0.967	19	0.59 (0.39, 0.91); 0.016
Exposure to HIV in the 6 months prior					
No	51	32	Ref	17	Ref
Yes	46	29	1.01 (0.7, 1.47); 0.95	25	1.64 (1.1, 2.47); 0.016
Employment					
Employed	51	29	Ref	20	Ref
Unemployed	42	34	1.39 (0.87, 2.23); 0.165	23	1.42 (0.86, 2.33); 0.173
Ever purchased products from an online pharmacy					
No	45	32	Reference	24	Reference
Yes	61	26	0.66 (0.42, 1.04); 0.071	13	0.42 (0.25, 0.71); 0.001
Currently taking PrEP					
No	51	29	Reference	20	Reference
Yes	37	37	2.08 (1.27, 3.41); 0.003	26	1.99 (1.17, 3.39); 0.011
Constant			0.83 (0.31, 2.27); 0.722		0.97 (0.32, 2.88); 0.95

^a^ Proportion of observations in each class for every characteristic such that each row adds up to 100%. Class 1: Prefer remote PrEP services (18.9% of the sample); Class 2: Prefer online PrEP with in‐person services (20.7%); and Class 3: Prefer online PrEP with remote services (60.3%).

^b^ RRR, relative risk‐ratios. RRR are estimated in a multivariate regression and are, therefore, adjusted by other variables. Ref, Reference.

The “prefer online PrEP with in‐person components” group (20.7%) placed the greatest importance on HIV test type, strongly preferring provider‐based testing over HIVST, with oral HIVST having the largest negative PW. Participants also preferred in‐person over remote clinical consultation; while they preferred guided assessment and call/SMS user support, this attribute had a small impact on decision‐making. Class membership was associated with currently taking PrEP and being male.

The “prefer remote PrEP” group (18.9%) had preferences similar to “prefer online PrEP with remote components” but was the only group with a positive opt‐out coefficient. However, the magnitude of the opt‐out coefficient was smaller than their preferences for remote services. Participants preferred remote consultation, online assessment and HIVST, with similar utility for both oral and blood‐based HIVST. They preferred phone user support, although this had the smallest impact on decision‐making. Group membership was associated with current PrEP use, HIV exposure in ≥6 months, completing secondary school or above and not previously made purchases from an online pharmacy. HIV‐test type followed by clinical consultation were the most important attributes for all three groups (See Appendix S3 for coefficients).

## 4 DISCUSSION

We evaluated preferences for a novel online pharmacy PrEP service among potential clients in Kenya. Online PrEP was highly acceptable both when assessed directly *via* questions based on the TFA and through the DCE (based on a large negative opt‐out PW). PrEP non‐users had a larger negative opt‐out coefficient than those currently taking PrEP, suggesting that online pharmacy PrEP could expand PrEP coverage to interested individuals whose preferences are not met by standard PrEP services. Overall, participants preferred remote clinical consultation, HIVST and phone/SMS user support; these attributes had a similar impact on decision‐making. Individuals also preferred online self‐administered eligibility assessment, although this had a lower relative impact on the choice of PrEP services. These results suggest that while participants value privacy when completing assessments regarding sexual and HIV risk behaviour, health and HIV status, however, when needing support for questions and concerns about PrEP, they prefer in‐person contact. This is consistent with qualitative studies of HIVST and emphasizes the importance of provider availability to support remote PrEP services [22]. Although blood‐based HIVST was preferred over oral HIVST in the overall sample, this pattern was not observed in the LCA, with most sub‐groups giving equal preference to both HIVST types. The difference observed in the overall sample is likely due to the strong negative preference for oral HIVST in the “prefer online PrEP with in‐person components” group, the only group that preferred provider‐administered HIV testing.

Despite the convenience of having a provider meet participants at home or in the community to administer HIV testing, this option was associated with a negative utility, with most participants preferring HIVST. This highlights the importance of privacy and autonomy over the testing process [23]. Studies from ESA find that barriers to HIV testing include distrust in providers to maintain confidentiality and perceptions of stigma and judgement from providers regarding high‐risk sexual behaviours [24]. HIVST can empower users by providing control over the testing process and ensuring confidentiality of results. Leveraging HIVST for community‐based PrEP provision has emerged as a promising strategy to increase PrEP uptake and retention. The SEARCH trial of dynamic HIV prevention showed that offering participants choices for HIV prevention services, including community‐based PrEP delivery and HIVST, substantially increased coverage of biomedical prevention during periods of HIV risk [25]. By the end of the trial, 65% of individuals in the intervention arm chose HIVST instead of provider‐administered testing for HIV prevention services and most chose community‐based delivery. Additionally, HIVST may facilitate PrEP provision in community settings by reducing the need for trained counsellors to administer testing. For example, in our team's recent trial of online pharmacy‐based PrEP provision in Kenya, participants received HIVST *via* courier to the setting of their choice, uploaded a photo of their HIVST result to a secure online platform and completed a telemedicine eligibility assessment. Those who were eligible could receive PrEP drugs from the online pharmacy. This service was highly acceptable and reached PrEP naïve populations at HIV risk [10]. One consideration is that HIVST has lower sensitivity than provider‐administered testing, potentially leading to inappropriate PrEP use among persons with undiagnosed HIV and the development of drug resistance. However, modelling studies found that HIVST use for PrEP provision would not meaningfully increase population‐level drug resistance and benefits of expanded PrEP coverage likely outweighed drug resistance risks [26, 27]. The WHO recently updated their guidance to support HIVST use for PrEP initiation and continuation [28].

When exploring preference heterogeneity, we found three sub‐groups in our sample. Most participants belonged to the “prefer online PrEP with remote components” (60.3%) whose preferences aligned with our main findings, namely remote clinical consultation, HIVST, phone/SMS user support and self‐administered eligibility assessment. These participants had a strong preference for online PrEP services (with a large negative opt‐out coefficient) and group membership was associated with not currently using PrEP, having previously used online pharmacies and being employed. These results suggest that online PrEP provision could reach persons who use online pharmacies and are interested in PrEP but not currently accessing it *via* clinics. The “prefer online PrEP with in‐person components” group (∼20% of the sample) also preferred online PrEP over their status quo but only when services contained a sufficient number of in‐person components, which include provider‐administered HIV testing, in‐person consultation, guided clinical assessment and phone/SMS user support (although the latter two characteristics were least important in decision‐making). Members in this group were more likely to be current PrEP users. This suggests that offering the option of increased provider interaction could expand online PrEP coverage to those who are interested but dislike fully virtual provision through remote/self‐conducted services. As current PrEP users are more likely to comprise this group, this suggests PrEP users may switch to online PrEP if it were made more interactive as it may be more convenient than clinic‐based PrEP. Finally, the “prefer remote PrEP” group (19% of the sample) preferred status quo but were willing to uptake online PrEP only if it is tailored to their preference for remote services, including remote consultation, HIVST and online assessment. Members of this group were more likely to be male and current PrEP users. Taken together, these findings highlight that the vast majority of participants prefer private and anonymous PrEP services with limited provider interaction unless initiated by a client for specific support needs. However, including flexible service options could increase uptake as a substantial minority of participants preferred online PrEP with in‐person components for PrEP initiation, although this group was more likely to be currently accessing PrEP at clinics. Our results are consistent with previous studies from low‐ and middle‐income countries that show high acceptability of remote clinical consultation and HIVST for PrEP service provision [29, 30] while underscoring the need for multiple options to accommodate varying preferences.

Telehealth is a promising strategy to reduce barriers to PrEP access, persistence and alignment with periods of HIV risk. Online PrEP provision can offer increased convenience, privacy and autonomy, resulting in greater coverage of diverse populations and geographies [31]. Although most telehealth PrEP evaluations have been conducted in high‐income settings, a study from Brazil found telehealth PrEP was associated with higher uptake and persistence compared to clinic‐based PrEP [32]. Telehealth platforms are rapidly expanding in Africa and growing online literacy results in a large population that can benefit from services. Improving digital literacy is a priority of the Kenyan government as only 17% of those in rural areas and 44% in urban areas have internet access [33, 34]. Online pharmacies are also growing in Africa and deliver a wide range of medicines and health products directly to consumers [35, 36]. These platforms often attract clients seeking discreet access to sexual and reproductive health products, including emergency contraception and HIVST, who may also be interested in PrEP [10]. Participants stated they were willing to pay $7 USD for online PrEP provision, which would enable private companies to charge for some service components. One caveat of PrEP telehealth services delivered via online pharmacies is that it largely reaches individuals at higher socio‐economic status who have internet access and computer/smartphone literacy [36]. While this platform can expand PrEP coverage to Kenya's growing middle class, further research is needed to explore telehealth modalities to serve individuals with lower socio‐economic status at risk of HIV.

Our study has several limitations. First, DCEs measure the hypothetical acceptability of services and not real‐world uptake. However, evidence evaluating the validity of health‐related DCEs found high accuracy in predicting user choices [37]. Nevertheless, it is not known to what extent our findings reflect actual uptake. Second, our recruitment approach relied on interested individuals contacting the study team via phone to enrol, therefore, we cannot estimate a denominator for acceptance rate. Additionally, participants were asked to complete an in‐person survey in a convenient location [8]. While a provider‐administered DCE likely resulted in higher data quality compared to an online self‐administered survey, individuals with greater privacy concerns may be underrepresented. However, despite this, we observed strong preferences for anonymous/remote services compared to in‐person options [38]. Third, we did not include a price attribute in the DCE so we could not measure marginal WTP for difference aspects of PrEP service. While piloting the DCE, we found that participants placed a large emphasis on the price attribute, which seemed to be a strong driver of decision‐making. To avoid having one attribute potentially overwhelm participant decisions, we removed the cost attribute from the DCE [8]. However, we assessed WTP directly through survey questions. Future research is needed to determine an appropriate price point that does not decrease demand while also producing sufficient revenue for sustainable services. This may require government subsidies implemented via partnerships between private online pharmacies and Ministries of Health.

Strengths of our study include a large and representative sample of potential online PrEP clients. We partnered with a Kenyan online pharmacy retailer to recruit individuals whose characteristics closely reflected the target population at HIV risk who were interested in online PrEP and had sufficient computer literacy to navigate a website. Most participants reported recent sexual behaviours associated with HIV risk including multiple partners, known exposure to HIV or STI treatment. Our large sample size enabled us to identify preference heterogeneity and evaluate interactions by current PrEP use. Additionally, the DCE included a dual opt‐out allowing us to estimate demand for online PrEP. To our knowledge, this is the first study to quantify user preferences for online pharmacy PrEP delivery in SSA.

## 5 CONCLUSIONS

Online PrEP is highly acceptable among potential users and may expand PrEP coverage to those interested in PrEP but not currently accessing services. Most participants valued privacy and autonomy, preferring HIVST, remote consultation, online self‐administered eligibility assessment and phone/SMS user support. Results can inform online pharmacy PrEP services to optimize uptake in Kenya and similar settings.

## COMPETING INTERESTS

The authors have no conflicts of interest to disclose.

## AUTHORS’ CONTRIBUTIONS

EMS, YC, MAM, MLM, KFO and MS conceptualized the study. All authors contributed to design, analysis plan, results interpretation and manuscript editing. EMS and YC conducted data analysis with mentorship from FT‐P. EMS, YC and MS wrote the first draft. NT, CK, MAM and KN led the implementation. All authors read and approved the manuscript and take responsibility for the decision to submit it for publication.

## FUNDING

The funding for this study was provided by Bill and Melinda Gates foundation. The grants were made to University of Washington (INV‐037646), MYDAWA (INV 035424), Jhpiego (INV‐035932) and Audere (INV‐038498).

## Supporting information




**Appendix S1**. Example choice task.
**Appendix S2**. Relative importance of attributes in the decision‐making process to acquire PrEP via online‐based services, for the overall sample and by class from the latent class analysis.
**Appendix S3**. Estimated coefficients for attribute‐levels for the overall sample and each class identified.

## Data Availability

The authors have provided the required Data Availability Statement, and if applicable, included functional and accurate links to said data therein.
