# Supplementary material for: Preferences for pre‐exposure prophylaxis delivery via online pharmacy among potential users in Kenya: a discrete choice experiment
Source: J Int AIDS Soc. 2024 Oct 9;27(10):e26356. doi: 10.1002/jia2.26356 (PMC11464213; doi:10.1002/jia2.26356)
Supplement: Supplementary file 1 — Appendix S1. Example choice task. Appendix S2. Relative importance of attributes in the decision‐making process to acquire PrEP via online‐based services, for the overall sample and by class from the latent class analysis. Appendix S3. Estimated coefficients for attribute‐levels for the overall sample and each class identified. [file JIA2-27-e26356-s001.docx]

# SUPPLEMENTAL MATERIAL

**Appendix 1.** Example choice task

| Which of these two options for online PrEP delivery would you most prefer | | | |
| --- | --- | --- | --- |
| (1 of 8 choice tasks) | | | |
| **Method for conducting client eligibility assessment for PrEP** | Online self-assessment using screening questions (phone number in case of questions)  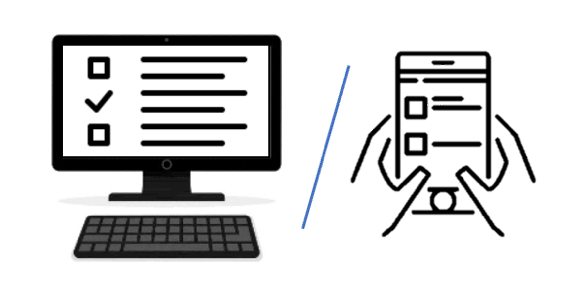 |  | Guided assessment with a remote clinical provider (via a phone call or WhatsApp)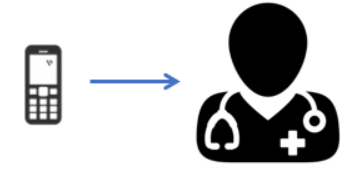 |
| **Type of HIV test delivered for PrEP initiation** | Healthcare provider administers HIV Test at setting of your choice (blood-based)  *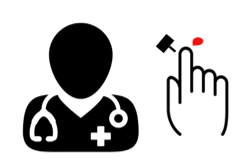* |  | Blood-based HIV self-test (at setting of your choice)  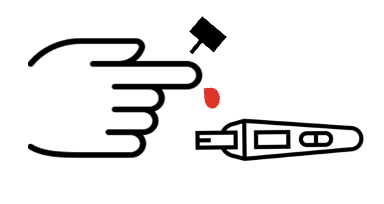 |
| **Clinical consultation needed to prescribe PrEP** | Remote clinical consultation with provider (via a phone call or video chat)  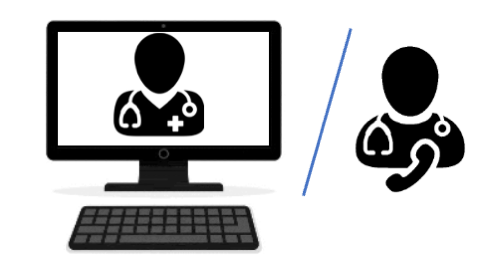 |  | In-person clinical consultation with provider after completing HIV test (at a setting of your choice)  *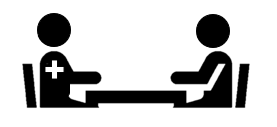* |
| **Method for discussing your questions for PrEP with a healthcare provider** | Email  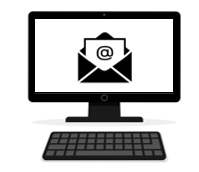 |  | Phone / video call  *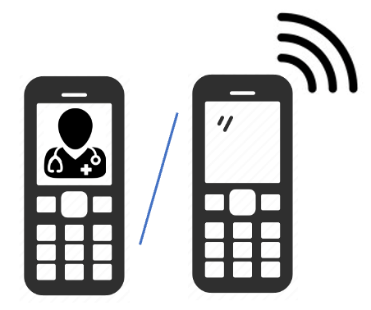* |
|  | Select |  | Select |
|  |  |  |  |
|  |  | | |
|  | If the PrEP service you just chose was available, do you think you would actually use it? | | |
|  | Yes |  | No |

**Appendix 2.** Relative importance of attributes in the decision-making process to acquire PrEP via online-based services, for the overall sample and by class from the latent class analysis.

**Appendix 3.** Estimated coefficients for attribute-levels for the overall sample and each class identified.

| **Attribute** | **Levels** | **Overall: PW (95%CI)** | **Class1: PW (95%CI)** | **Class2: PW (95%CI)** | **Class3: PW (95%CI)** |
| --- | --- | --- | --- | --- | --- |
| Assessment | Online self-applied | 0.04  (0.01, 0.07) | 0.39 (0.3, 0.48) | -0.07 (-0.12, -0.01) | 0.03 (-0.01, 0.07) |
|  | Guided | -0.04  (-0.07, -0.01) | -0.39 (-0.48, -0.3) | 0.07 (0.01, 0.12) | -0.03 (-0.07, 0.01) |
| Test | Oral self | 0.02  (-0.04, 0.07) | 0.61 (0.48, 0.73) | -0.6 (-0.71, -0.49) | 0.21 (0.13, 0.29) |
|  | Blood self | 0.15  (0.1, 0.19) | 0.7 (0.58, 0.83) | -0.18 (-0.28, -0.09) | 0.22 (0.15, 0.29) |
|  | Provider-based | -0.16  (-0.23, -0.09) | -1.31 (-1.48, -1.14) | 0.79 (0.67, 0.91) | -0.43 (-0.52, -0.34) |
| Consultation | Remote | 0.13  (0.09, 0.18) | 0.94 (0.81, 1.06) | -0.5 (-0.57, -0.43) | 0.3 (0.24, 0.36) |
|  | In-person | -0.13  (-0.18, -0.09) | -0.94 (-1.06, -0.81) | 0.5 (0.43, 0.57) | -0.3 (-0.36, -0.24) |
| User Support | SMS | 0.08  (0.04, 0.12) | 0.04 (-0.07, 0.15) | 0.08 (-0.01, 0.17) | 0.14 (0.07, 0.2) |
|  | Call | 0.11  (0.07, 0.15) | 0.14 (0.03, 0.25) | 0.08 (-0.01, 0.17) | 0.14 (0.07, 0.21) |
|  | Email | -0.19  (-0.24, -0.14) | -0.18 (-0.29, -0.07) | -0.16 (-0.25, -0.06) | -0.28 (-0.35, -0.21) |
| Opt-Out | Opt-out | - | 0.71 (0.58, 0.84) | -0.26 (-0.42, -0.11) | -4.01 (-4.53, -3.48) |
|  | Opt-out: non-PrEP Users | -1  (-1.14, -0.86) | - | - | - |
|  | Opt-out: PrEP Users | -0.5  (-0.75, -0.25) | - | - | - |

Abbreviations. CI: confidence interval; PW: Preference Weights.

Description of each class: 1) Class 1: Prefer remote PrEP services (18.9% of sample), 2) Class 2: Prefer online PrEP with in-person services” (20.7%), and 3) Class 3: Prefer online PrEP with remote services” (60.3%).
